# Determinants of uterine synechiae at hysteroscopy: a case-control study in Kinshasa, Democratic Republic of Congo

**DOI:** 10.11604/pamj.2025.51.64.43555

**Published:** 2025-07-03

**Authors:** Emmanuel Nzau-Ngoma, Armand Lusakueno Lumingu, Patrick Moleko Mindombe, Bruno Lusila Biawila, Amos Kusuman, Jules Mpoy Odimba

**Affiliations:** 1 Department of Gynecology and Obstetrics, University Clinics of Kinshasa, Kinshasa, Democratic Republic of Congo,; 2 Endo-conception Clinic, DAEMMI Berlinde foundation, Kinshasa, Democratic Republic of Congo

**Keywords:** Synechiae, determinants, hysteroscopy

## Abstract

In most cases, synechiae originate from traumatic uterine procedures in a gravid or non-gravid uterus. The aim of this study was to identify determinants of uterine synechiae in a population of Kinshasa. A case-control study was conducted at the Endo-conception Clinic in Kinshasa, DRC, from March 1^st^, 2018, to February 28^th^, 2023. Cases were patients with uterine synechiae in hysteroscopy, and controls were patients with normal hysteroscopy. The comparison of proportions was made using the Pearson Chi-square test and Fisher's Exact. The student’s t-test was used to compare means whose variables were normally distributed and the comparison of the medians of parity and gravidity was made with the U Mann-Whitney test. The test was significant for a p-value < 0.05. A total of 223 patients with synechiae (cases) and 472 patients with normal hysteroscopy (controls) were collected. The mean age was 38.41 years ± 6.60 for cases and 36.91 years ± 6.86 for controls. Comparing cases to controls, history of curettage was present in 69.1% versus 37.3 %, history of uterine surgery in 58.7% versus 35.6% and dysmenorrhea in 17.9% versus 8.9%. Factors associated with uterine synechiae were dysmenorrhea (aOR: 2.51, 95% CI 1.54 - 4.19; p=0.000), history of curettage (aOR: 3.32, 95% CI 1.97 - 5.60; p = 0.000) and uterine surgery (aOR: 2.96, 95% CI 2.08 - 4.21; p = 0.000). The findings demonstrate a notable frequency of uterine synechiae in patients with a history of curettage, uterine surgeries, and dysmenorrhea in cases compared to controls, identifying them as determinants of the condition.

## Introduction

**Figure 1:**
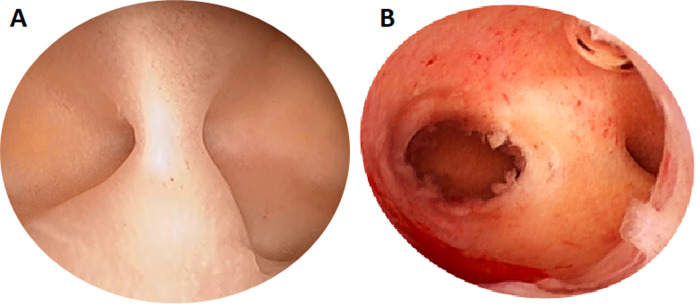
photographs of partial synechiae: A) fibrous band just in front of the right tubal orifice; B) median fibrous band spread slightly laterally (source: Endoconception Clinic/FoDAB, DRC)

**Figure 2:**
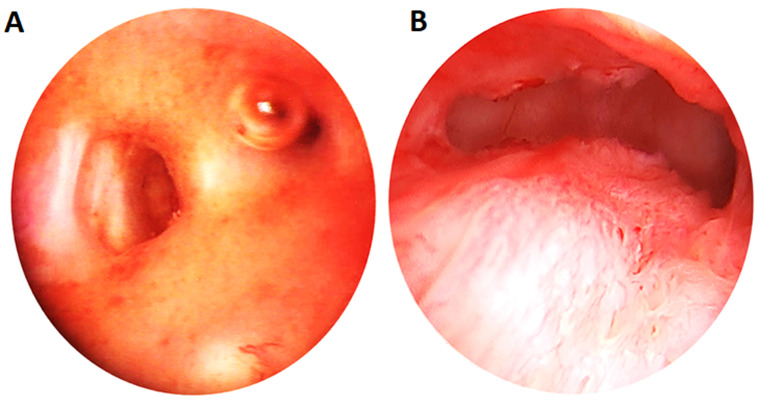
A) tip of the hysteroscope is at the isthmic level and a fibrous band is noted closing the uterine cavity with cul-de-sac openings; B) beyond the cervical canal, a cul-de-sac is noted isthmic testifying to total synechiae (source: Endoconception Clinic/FoDAB)

Uterine synechiae are intra-uterine adhesions characterized by partial or total adhesion of the internal uterus walls at whatever location it could be, from the external orifice of the cervix to the fundus of the uterus [1]. They are partial when they affect only a variable fraction of the uterine cavity and/or the cervix, where the internal orifice of the cervix is generally visible (Figure 1). Cases that are said total involve the corporal cavity as a whole and the uterine isthmus, with the internal orifice of the cervix that is almost invisible, and the fibrous bands take up all the corporal cavity and sometimes the uterine isthmus (Figure 2) [2].

In most cases, synechiae are asymptomatic and diagnosed during a routine infertility work-up [3]. When symptomatic, they can entail troubles such as amenorrhea or hypomenorrhea, chronic pelvic pain, infertility, recurrent spontaneous miscarriage, and placentation anomalies [4,5]. The frequency of uterine synechia varies in the world, depending on the populations and the settings concerned [2]. In Cameroon, Nyada *et al*. [6] found 20.8% of synechiae in patients coming for in-vitro fertilization (IVF). We reported a similar proportion (20.5%) in the Democratic Republic of Congo [7], in patients who underwent hysteroscopy for confounded indications.

Its consequences include infertility [8], repeated spontaneous abortions and significant obstetric morbidity in terms of abnormal placentation (prematurity, intrauterine growth retardation, placenta accreta, placenta previa) [3,8].

The prevalence of synechiae varies according to numerous risk factors [3] and the type of population studied, but pregnancy remains unquestionably the most important risk factor [9]. Around 95% of synechiae form in the post-abortum or post-partum period because of endometrial trauma [10,11]. Apart from pregnancy, other factors may also be involved, although they are less frequently responsible for uterine synechiae. These include abortion, particularly clandestine abortion with curettage, the method of evacuation of post-partum and post-abortal retentions, genital tuberculosis, and post-partum infections [8,12,13]. Constitutional factors, different uterine surgical techniques, and certain uterine compression methods may also cause uterine synechiae [14,15]. Vascular disorders, menopause, and thermal aggression are also mentioned as etiological factors in uterine synechiae [15,16]. Given the variability of risk factors among different settings, their acknowledgment in a particular area is the key step to planning for prevention. This study aimed to describe the socio-demographic and clinical characteristics and identify the determinants of uterine synechiae during hysteroscopy in a population of Kinshasa, the capital of the Democratic Republic of Congo.

## Methods

**Study design and setting:** this was a case-control study which took place at “Endo-conception Clinic” in Kinshasa, a private practice clinic receiving patients from all around the city. The study focused on the period from March 1^st^, 2018 to February 28^th^, 2023. Cases were determined by the presence of uterine synechiae diagnosed at hysteroscopy and controls were characterized by the absence of any pathology at hysteroscopy (normal hysteroscopy).

**Study population:** the study population consisted of patients who underwent hysteroscopy regardless of the indication during the study period. Records of patients presenting with uterine synechiae in hysteroscopy during the study period were included as cases (n =223), and those with normal hysteroscopy (n=472) were included as controls. It has been considered the "placental retention" as the main exposure and the diagnosis of uterine synechiae as the event [17]. With a 5% type 1 risk, a 90% power, a 15.03% of synechiae in patients with the main exposure and 6.7% patients with the main exposure without synechiae, a least extreme odds ratio of 2.35 (70) and a distribution of cases/controls of 0.5, the calculated sample size was 214 patients. And since our sample had 223 cases, we retained them all, and the control group had 472 patients.

**Data collection:** the data were collected from the files of patients who underwent hysteroscopy during the study period. We used an elaborate form to extract data from the record. The socio-demographic variables were the age, the marital status, and the level of education. The clinical variables were parity, gravidity, presence of dysmenorrhea, history of abortion/miscarriage, curettage, uterine surgery, and presence of synechiae at hysteroscopy.

**Statistical analysis:** an electronic form was used to record data using EPI data 3.1 software. The database from EPI data was exported to Social Package for Social Sciences 23.0 software for analysis. Qualitative variables were presented as of proportions (%) and quantitative ones as mean and standard deviation or median and extremes (minimum and maximum), depending on the case. Comparison of proportions between groups was made using Pearson's chi-square test or Fisher's exact test. The Student's t-test was used to compare means for age between groups, and the Mann-Whitney U-test was used to compare medians for parity and gravidity. Logistic regression in bi and multivariable analyses using the “enter” method was used to generate the Odds Ratio to measure the strength of the association between the dependent variable and the independent variables. The test was significant for a p-value < 0.05.

**Ethical considerations:** the project of this study was approved by the Ethics Committee of the Kinshasa School of Public Health (approval notice: ESP/CE/135/2022).

## Results

**Figure 3:**
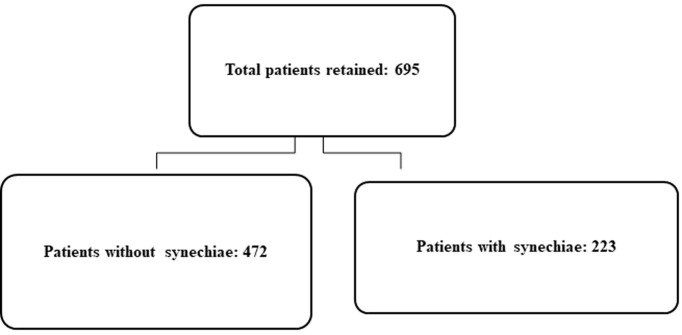
patients flow chart

During the present study, we listed a total of 695 hysteroscopies with either normal (472) or uterine synechiae findings (223) (Figure 3). The mean age was 38.41 years ± 6.60 for cases and 36.91 years ± 6.86 for controls, and the difference was statistically significant (p = 0.006). There was no statistical difference considering marital status (0.085) and level of education (p = 0.539) (Table 1).

**Table 1:** distribution of patients according to their sociodemographic characteristics

Variables				p-value
	Case (n=223) n (%)	Control (n=472) n (%)	Total n (%)	
**Age**				
Mean ± standard deviation	38.41±6.60	36.91±6.86	37.39 ± 6.81	0.006
< 25 years	3 (1.3%)	18 (3.8%)	21 (2.5)	
25-34 years	62 (27.8%)	152 (32.2%)	214 (30)	
35-44 years	123 (55.2%)	235 (49.8%)	358 (52.5)	
≥ 45 years	35 (15.7%)	67 (14.2%)	102 (15)	
**Marital status**				**0.085**
Single	47 (21.1%)	72 (15.3%)	119 (18.2)	
Divorced	6 (2.7%)	5 (1.1%)	11 (1.9)	
Married	169 (75.8%)	392 (83.1%)	561 (79.4)	
Widow	1 (0.4%)	3 (0.6%)	4 (0.5)	
**Level of education**				**0.539**
Primary	2 (0 .9%)	5 (1.1%)	7 (1)	
Secondary	47 (21.1%)	83 (17.6%)	131 (19.3)	
University	174 (78%)	384 (81.3%)	560 (79.7)	

In the case-group, 69.1% of patients had a history of curettage compared to 37.3% in the control-group (p = 0.000). Dysmenorrhea was present in 17.9% of cases compared to 8.9% of controls (p=0.001). In a similar trend, 58.7% of cases had a history of uterine surgery compared to 35.6% in controls (p=0.000). The types of uterine surgeries that patients underwent in the past were homogeneously distributed in the two groups (p = 0.239), although laparotomic myomectomy represented 71.6% of all procedures (Table 2).

**Table 2:** distribution of patients according to their gynecological and obstetrical history

Variables	Case (n= 223) n (%)	Controls (n= 472) n (%)	Total n (%)	p-value
**Parity**				
Median (min, max)	0 (0.5)	0 (0.7)	0 (0.7)	0.007
Nulliparous	132 (59.2%)	294 (62.3%)	426 (60.8)	
Pauciparous	79 (35.4%)	125 (26.5%)	204 (30.9)	
Multiparous	12 (5.4%)	53 (11.2%)	65 (8.3)	
**Gravidity**				
Median (min, max)	2 (0.10)	1 (0.11)	2 (0.1)	0.000
Nulligravida	39 (17.5%)	146 (31.0%)	185 (24.2)	
Paucigravida	84 (37.7%)	188 (39.8%)	272 (38.8)	
Multigravida	100 (44.8%)	138 (29.2%)	238 (37.0)	
**Notion of dysmenorrhea**				**0.001**
No	183 (82.1%)	430 (91.1%)	613 (86.6)	
Yes	40 (17.9%)	42 (8.9%)	82 (13.4)	
**History of uterine surgery**				**0.000**
No	92 (41.3%)	304 (64.4%)	396 (52.8)	
Yes	131 (58.7%)	168 (35.6%)	299 (47.2)	
**Type of uterine surgery (n=299)**				**0.239**
Laparotomic myomectomy	90 (68.7)	124 (73.8)	214 (71.6)	
Cesarean section	23 (17.6)	31 (18.5)	54 (18.1)	
Operative hysteroscopy	18 (13.7)	13 (7.7)	31 (10.4)	
**History of curettage**				**0.000**
No	69 (30.9%)	296 (62.7%)	365 (46.8)	
Yes	154 (69.1%)	176 (37.3%)	330 (53.2)	

In univariable analysis, factors associated with uterine synechiae were: multigravidity, presence of dysmenorrhea, history of abortion/miscarriage, history of uterine surgery, and history of uterine curettage. In multivariable analysis, only dysmenorrhea (aOR: 2.51, 95% CI 1.54 - 4.19; p=0.000), history of curettage (aOR: 3.32, 95% CI 1.97 - 5.60; p = 0.000) and of uterine surgery (aOR: 2.96, 95% CI 2.08 - 4.21; p = 0.000) emerged as determinants of uterine synechiae (Table 3).

**Table 3:** factors associated with uterine synechiae in patients who have undergone hysteroscopy

Variables	Risk of uterine synechiae			
	Unadjusted OR (95%CI)	p-value	Adjusted OR (95%CI)	p-value
**Multigravidity**				
Yes	1.97 (1.41-2.74)	0.000	1.27 (0.86-1.88)	0.229
**Nulliparity**				
Yes	0.88 (0.63-1.22)	0.434		
**Abortion**				
Yes	2.93 (2.02-4.25)	0.000	1.14 (0.63-2.07)	0.659
**Curettage**				
Yes	3.75 (2.67-5.27)	0.000	3.32 (1.97-5.60)	0.000
**Dysmenorrhea**				
Yes	2.24 (1.40-3.57)	0.001	2.51 (1.51-4.19)	0.000
**Uterine surgery**				
Yes	2.58 (1.86-3.57)	0.000	2.96 (2.08-4.21)	0.000

## Discussion

The objectives of the current study were to describe the socio-demographic and clinical characteristics and to identify the determinants of uterine synechiae in a population of Kinshasa in the Democratic Republic of Congo. Patients were mostly in the reproductive age with the mean ages of 38.41 years ± 6.60 and 36.91 years ± 6.86 for cases and controls, respectively. The history of curettage, of uterine surgery, and dysmenorrhea were most prevalent in cases compared to controls and were determinants of uterine synechiae. According to the age in both groups, the findings in the present study are in agreement with those made by Daaloul *et al*. [18] in Tunisia and Capmas *et al*. [19] in France, which were 35.4 and 34.3 years, respectively. Whereas it differs from that reported by Goldenberg *et al*. [20] in Israel, who noted 28.5 years. This difference may be explained by the fact that, in the present study, the patients included as cases had only synechiae, whereas in the study by Goldenberg *et al*. [20], they had synechiae and uterine septa [21]. Since septal defects are a congenital pathology, it is understandable that patients with uterine septal symptoms were relatively young.

The risk of finding synechiae in patients with dysmenorrhea was multiplied by 2. On the one hand, this finding could be justified by the high prevalence of genital infections and the precariousness of post-abortum and post-partum care (source of endometritis) in our settings [22,23]. On the other hand, dysmenorrhea in patients with uterine synechiae could suggest endometriotic lesions secondary to synechiae or adenomyosis. Indeed, endometriotic lesions appear to be the result of increased menstrual reflux from the tubes due to the block to the vaginal flux [2]. A history of curettage quadrupled the risk of uterine synechiae. This result is in line with those found in the literature [24,25]. One fact that was demonstrated in this study is that abortions, when taken alone (bivariate analysis), showed an association with uterine synechiae. However, it is the "curettage" character (multivariate analysis) that emerges as the determinant of synechiae. In fact, the risk of uterine synechiae associated with curettage may be aggravated by certain factors, notably increased endometrial trauma (highly abrasive), post-abortum infections, and certain vascular disorders (thrombosis) [11,26]. In this study, the risk of finding uterine synechiae at hysteroscopy was multiplied by 3 in patients with a history of uterine surgery of any type. This result is in accordance with those reported in the literature [27-29]. In their study of hysteroscopy after laparotomic myomectomy, Dubuisson *et al*. [29] noted 35.6% of synechiae after uterine surgery. As for Tulandi *et al*. and Hurst *et al*. [27,28], 90% of synechiae were found in patients after laparotomic myomectomy. This disparity in proportions may be explained by the fact that many published studies are retrospective, and comparison is difficult due to the heterogeneity of the populations studied. In addition, in our setting, certain factors may explain this increased risk of synechiae, notably myoma topography (classes 0, 1, 2), myomas located on opposite sides of the uterine walls [2], and the size and number of enucleated myomas [30]. Factors linked to individual predisposition [31] and the environment [32] may also explain the high risk of adhesions in the case of laparotomic myomectomy. This risk is the greatest when the myoma is posterior and interstitial [33-35].

The combination of uterine surgery and uterine synechiae should prompt the practitioner to take much greater care when caring for women with myomas to be operated on or already operated on, as these women should be considered at risk. It is therefore important to implement measures to prevent intrauterine adhesions, including rigorous asepsis, strict application of microsurgical principles, and the use of antiadhesion barriers, as well as second-look hysteroscopy between the sixth and eighth post-operative weeks, depending on the team.

This study, being in a hospital setting, only concerned records of patients of all categories who benefited from diagnostic hysteroscopy during the study period at the Endo-conception Clinic in the city of Kinshasa. It is therefore not representative of the general population of our country (DRC). As a result, their socio-demographic, clinical characteristics, and intraoperative findings cannot be extrapolated to the general population. The fact of not having the histopathological nature of uterine synechiae for the American Fertility Society (AFS) classification of synechiae was also a limitation to this study. In the end, the fact that we used the patients' files did not allow us to have all their general characteristics.

## Conclusion

Uterine synechiae are more likely to be diagnosed in patients of reproductive age. They are mostly nulliparous and multi-gravida with menstrual disorders, particularly dysmenorrhea. The risk factors associated to uterine synechiae are curettage, uterine surgery, and dysmenorrhea. The strong association with prior uterine surgery, particularly in a setting where open myomectomy is prevalent, suggests a need for tailored preventative measures during and after these procedures. Furthermore, the independent association of dysmenorrhea with synechiae warrants further investigation into potential shared inflammatory pathways within this population. These findings emphasize the need for targeted interventions, such as modified surgical techniques and proactive management of dysmenorrhea, to reduce the burden of uterine synechiae in Kinshasa. Future research should explore the specific surgical techniques contributing to synechiae formation and the underlying pathophysiology linking dysmenorrhea and intrauterine adhesions in this population.

### What is known about this topic

- Endouterine trauma on a pregnant uterus is the main etiology of uterine synechiae;
- Uterine synechiae can lead to infertility, recurrent pregnancy loss, and menstrual abnormalities;
- Currently, hysteroscopy is the reference technique for both diagnosis and treatment of synechiae.

### What this study adds

- In patients presenting with the triad: dysmenorrhea, history of curettage, and history of uterine surgery, the diagnosis of uterine synechiae should be evoked;
- Dysmenorrhea is an independent risk factor for uterine synechiae, suggesting a potential link between chronic inflammation or endometriosis and the development of adhesions;
- The study provides specific risk factors of uterine synechiae in the Kinshasa, Democratic Republic of Congo population, which may differ from those in other regions.
